# Editorial: Early Life Stress and Developmental Programming of Immune and Nervous System Responsivity

**DOI:** 10.3389/fcell.2022.897251

**Published:** 2022-04-14

**Authors:** James A. Coffman, Michael A. Burman, Erik R. Duboué

**Affiliations:** ^1^ Mount Desert Island Biological Laboratory, Bar Harbor, ME, United States; ^2^ University of New England, Portland, ME, United States; ^3^ Florida Atlantic University, Boca Raton, FL, United States

**Keywords:** immune system, central nervous system, early life stress, developmental programming, stress responsivity

Over the past few decades, it has become increasingly clear that early life experiences can have broad effects on development that have long term consequences on physiology and behavior. For example, early life stress (ELS) can have persistent developmental effects that impact how the body responds to stressors later in life. Such “developmental programming” has been linked to increased risk of developing metabolic and inflammatory diseases, behavioral problems, and mental illness. The programming appears to involve a complex interplay between the central nervous system, endocrine system, immune system, and metabolism, persistently affecting physiological set points and gene regulation with functional consequences on homeostasis throughout the body. Often, such effects become encoded in the epigenome and are passed into subsequent generations. One central challenge for the field has been elucidating the molecular/genetic and cellular regulatory circuitry underlying stress-induced developmental programming. The six papers (four research articles and two reviews) collected in this Research Topic take a broad approach to this problem by presenting findings from a variety of early life stressors, using vertebrate model organisms (rodent and zebrafish). These papers address the following specific questions:

1) How do different forms of neonatal maternal deprivation (daily chronic vs. random episodic) stress affect spatial learning in puberty (Stoneham et al.)
2) How does prenatal stress affect adult behavior related to depression and anxiety, and gene expression linked to the excitatory/inhibitory balance in the prefrontal cortex and amygdala (Marchisella et al.)
3) How does neonatal pain impact corticotropin-releasing factor (CRF) signaling in the amygdala leading to later life behavior dysfunction (Davis et al.)
4) Does ELS increase susceptibility to migraines and its neuroimmune underpinnings, and is this mitigated by exercise (Eller et al.)
5) What advantages and insights do zebrafish offer as a model for studies of developmental programming in response to ELS (Eachus et al.; Gans and Coffman)
6) How does glucocorticoid signaling contribute to stress-induced developmental programming (Gans and Coffman; Eachus et al.)

More broadly, these papers highlight some thematic issues associated with the problem of developmental/epigenetic programming in response to ELS. These include the existence of different developmental stage-specific windows conferring vulnerability to exposure and/or plasticity enabling therapeutic intervention, and the need to account for cell-, tissue-, sex-, species-, and genotype-specific effects of ELS. Both issues highlight the complex interplay between environment, genetics, and development, and thus the dangers inherent in generalizing from individual studies and populations. Nevertheless, this topic also highlights the advantages of comparative studies involving multiple systems for identifying robust commonalities among diverse systems, helping elucidate the general principles that will ultimately inform our understanding of ELS-induced developmental programming.

Another theme that emerges from these papers concerns the central influence of early life events in determining the function of the hypothalamus-pituitary-adrenal (HPA) axis in regulating the body’s response to subsequent stressors. Activation of this system is governed by neural systems including the amygdala and pre-frontal cortex, which are themselves altered by ELS. The HPA axis, in turn, regulates production of adrenal glucocorticoid hormones (cortisol in humans and zebrafish, and corticosterone in rodents), which affects gene expression in, and therefore function of, most cells in the body, including those of the immune system, central nervous system, liver, muscle, and bone. In addition, CRF is a key regulator of cellular responses in brain structures important for behavioral and emotional regulation. Finally, transcriptional effectors of glucocorticoid signaling such as the glucocorticoid receptor are known to mediate epigenomic remodeling. Thus, by perturbing activity and development of the HPA axis, and the systems that control it, ELS can have profound and persistent effects on the entire stress response system.

Finally, the variety of approaches and models described in this collection highlights the multifaceted complexity of the problem, which demands multi-disciplinary approaches to gain a systems-level understanding of the myriad ways in which ELS interacts with development, and how that impacts the various molecular, cellular, genetic, and epigenetic mechanisms involved in establishing homeostatic set points governing stress responsivity across systems. While this is a burgeoning field, it is still very much in its infancy and there is much to be learned from a variety of model systems as well as human epidemiology and clinical studies.

